# A recombinant Modified Vaccinia virus Ankara expressing prME of tick-borne encephalitis virus affords mice full protection against TBEV infection

**DOI:** 10.3389/fimmu.2023.1182963

**Published:** 2023-04-21

**Authors:** Mareike Kubinski, Jana Beicht, Isabel Zdora, Jeannine Biermann, Christina Puff, Thomas Gerlach, Alina Tscherne, Wolfgang Baumgärtner, Albert D. M. E. Osterhaus, Gerd Sutter, Chittappen Kandiyil Prajeeth, Guus F. Rimmelzwaan

**Affiliations:** ^1^ Research Center for Emerging Infections and Zoonoses, University of Veterinary Medicine Hannover, Foundation, Hannover, Germany; ^2^ Department of Pathology, University of Veterinary Medicine Hannover, Foundation, Hannover, Germany; ^3^ Center for Systems Neuroscience, Hannover Graduate School for Neurosciences, Infection Medicine, and Veterinary Sciences (HGNI), Hannover, Germany; ^4^ Division of Virology, Institute for Infectious Diseases and Zoonoses, Ludwig Maximilian University Munich, Munich, Germany; ^5^ German Center for Infection Research (DZIF), partner site Munich, Munich, Germany

**Keywords:** TBEV, MVA, FSME-IMMUN^®^, protection, vaccination, virus-neutralizing antibodies, T cells

## Abstract

**Introduction:**

Tick-borne encephalitis virus (TBEV) is an important human pathogen that can cause a serious disease involving the central nervous system (tick-borne encephalitis, TBE). Although approved inactivated vaccines are available, the number of TBE cases is rising, and breakthrough infections in fully vaccinated subjects have been reported in recent years.

**Methods:**

In the present study, we generated and characterized a recombinant Modified Vaccinia virus Ankara (MVA) for the delivery of the pre-membrane (prM) and envelope (E) proteins of TBEV (MVA-prME).

**Results:**

MVA-prME was tested in mice in comparison with a licensed vaccine FSME-IMMUN® and proved to be highly immunogenic and afforded full protection against challenge infection with TBEV.

**Discussion:**

Our data indicate that MVA-prME holds promise as an improved next-generation vaccine for the prevention of TBE.

## 1 Introduction

Tick-borne encephalitis virus (TBEV) is a member of the family *Flaviviridae* and is an important emerging zoonotic pathogen, mainly transmitted by ticks, and responsible for up to 15,000 clinical cases in Europe and Asia annually (1). The number of tick-borne encephalitis (TBE) cases in several European countries is increasing (2, 3), and the geographical spread of TBEV is expanding (4–6). There are three main subtypes of the virus, the European, Siberian, and Far-Eastern, which differ in the severity of associated disease, geographical spread, and transmitting tick species (7).

TBEV has a positive-sense, single-stranded RNA genome with one open reading frame. The polyprotein is co- and post-translationally cleaved by viral and host proteases into three structural (C: capsid; prM: pre-membrane; E: envelope) and seven non-structural (NS) proteins (NS1, NS2A, NS2B, NS3, NS4A, NS4B, NS5). The E protein has several functions during the TBEV life cycle including receptor binding and entry into host cells. Since it is a target for virus-neutralizing (VN) antibodies, it is also important for the induction of protective immunity (8).

After TBEV infection, disease progression in humans can vary depending on viral (subtype, virulence, infection dose) and host factors (age, immune and health status, genetics). Infection with the European subtype of TBEV is mostly asymptomatic. In case of a symptomatic infection, patients develop mainly a biphasic disease. After mild, non-specific symptoms like fever and headache, an asymptomatic period follows which can develop into a second phase with neurological symptoms (e.g., meningitis, meningoencephalitis, meningoencephalomyelitis) also known as TBE. Some patients may have long-lasting sequelae, and in rare cases, TBEV infection can be fatal (7, 9). In Russia and Kazakhstan, specific immunoglobulins are given to patients who contracted a tick bite (7). However, in Europe, no antiviral drugs against TBEV are available. Hence, TBE-associated symptoms can be alleviated by supportive treatment only (7, 9). The most important protective measure against TBEV infection is vaccination. Globally, six TBE vaccines have been licensed, all based on inactivated TBEV preparations. Immunization regimens with TBE vaccines are time-consuming because after a primary round of three immunizations, regular booster vaccinations are recommended to maintain long-lasting protection (10). Vaccination with TBE vaccines induces protective antibodies, mainly against E, and CD4^+^ T cells against C and E. In contrast, natural infection with TBEV induces protective antibodies against E and NS1 as well as CD4^+^ (against C, E, and NS1) and CD8^+^ T cells (against NS2A, NS3, NS4B, and NS5) (10). Although the use of the licensed TBE vaccines results in high seroconversion rates (11–13) and is highly effective (14), they fail to afford complete protection against TBEV infection. Reports of breakthrough infections in fully immunized patients are consistently reported (15–18), and some of these cases even have a fatal outcome (19, 20).

A disadvantage of inactivated vaccines is that inactivation with formalin can result in antigenic modulation of viral epitopes, resulting in impaired induction of VN antibodies as has been shown for TBEV (21, 22). Therefore, the delivery of the native protein by using, e.g., viral vaccine vectors, may result not only in the induction of effective VN antibodies but also of potent CD4^+^ and CD8^+^ T cell responses (23) and should therefore be considered an attractive approach for the development of improved vaccines. Modified Vaccinia virus Ankara (MVA) is a highly attenuated poxvirus which was successfully used previously as a viral vector for vaccination and therapeutic approaches. MVA was generated by extensive passaging in primary chicken embryo fibroblasts (CEFs) which had led to the loss of large parts of its genome including factors important for virulence, pathogenesis, and virus–host interactions (24). Consequently, MVA is highly attenuated in human cells and can be also used for persons at risk like immunocompromised individuals (25–27). The safety and immunogenicity of MVA-based vaccines against a variety of viral pathogens, including Middle East respiratory syndrome coronavirus (MERS-CoV), severe acute respiratory syndrome coronavirus type 2 (SARS-CoV-2), influenza A virus (IAV), cytomegalovirus (CMV), and human immunodeficiency virus (HIV), have been demonstrated in clinical trials (28–34).

In the present study, we generated and evaluated a recombinant MVA that drives the expression of the prM and E genes of TBEV Neudoerfl (European TBEV subtype; MVA-prME). Previously, E protein-based vaccine candidates have been shown to induce VN antibodies and CD4^+^ T cells. However, the protective efficacy of these candidates was tested in a few studies only, and information on the induction of virus-specific CD4^+^ and CD8^+^ T cell responses is sparse (10). After *in vitro* characterization of MVA-prME, its ability to induce virus-specific antibody and T cell responses was investigated in mice. Furthermore, the protective efficacy of MVA-prME immunization against a lethal challenge infection with TBEV Neudoerfl was tested in mice. The results obtained with MVA-prME were compared with those obtained with the licensed vaccine FSME-IMMUN^®^. Based on our findings, it was concluded that MVA-prME holds promise as a novel next-generation TBE vaccine.

## 2 Materials and methods

### 2.1 Viruses and cells

Non-recombinant MVA F6 (MVA) and MVA expressing green fluorescent protein (GFP) under transcriptional control of the Vaccinia virus (VACV) late promoter P11 in deletion site III (MVA-GFP) were obtained from the Institute for Infectious Diseases and Zoonoses, Ludwig Maximilian University (LMU) Munich, Munich, Germany. TBEV strain Neudoerfl (European subtype) was obtained from the Department of Microbiology of the German Armed Forces, Munich, Germany. Primary CEF cells were produced from 10- to 11-day-old chicken embryos (specific pathogen-free eggs were purchased from Osterholz-Scharmbeck, Germany) and cultured in Minimum Essential Medium Eagle (MEM, Sigma-Aldrich, St. Louis, Missouri, USA) supplemented with 10% fetal bovine serum (FBS, heat-inactivated), 1% penicillin–streptomycin (Pen/Strep, Sigma-Aldrich, St. Louis, Missouri, USA), and 1% MEM non-essential amino acid solution (MEM NEAA, Sigma-Aldrich, St. Louis, Missouri, USA). A549 cells were maintained in F-12 Nut Mix (1×) + GlutaMAX^TM^, 10% FBS, 1% Pen/Strep, 1% GlutaMAX™, and 20 mM HEPES. HeLa cells were cultured in Dulbecco’s Modified Eagle Medium supplemented with 10% FBS, 1% Pen/Strep, 1% MEM NEAA, and 1% GlutaMAX^TM^. All cells were maintained at 37°C in a humid atmosphere with 5% CO_2_. Materials were purchased from Gibco™ (Waltham, Massachusetts, USA) unless otherwise stated. Used infection media were based on the respective maintaining medium with 2% FBS only. Viruses used for animal studies, A549 and HeLa cells were tested negative for mycoplasma (InvivoGen, San Diego, California, USA).

### 2.2 Generation of MVA-prME

**Figure 1:**
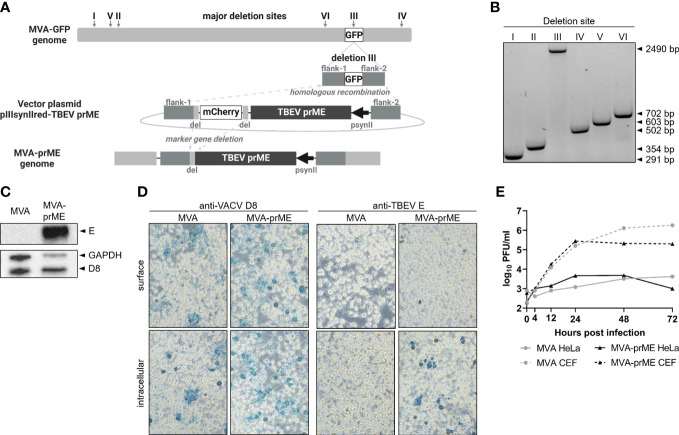
Generation of MVA-prME and *in vitro* characterization. **(A)** Homologous recombination within deletion site III of MVA-GFP and pIIIsynIIred-TBEV prME as well as intragenomic homologous recombination (marker gene deletion) generated recombinant MVA expressing prME of TBEV (MVA-prME). Created with BioRender.com. **(B)** Separation of DNA on 1% agarose TBE gel amplified by PCRs targeting the six major deletion sites of MVA (I: 291 bp, II: 354 bp, III: 447 bp, IV: 502 bp, V: 603 bp, VI: 702 bp). Successful integration of prME in deletion site III was verified (III: 2,490 bp). **(C)** Expression of TBEV E protein demonstrated by Western blot on MVA-prME-infected HeLa cells (MOI 5, 24 hpi). For controls, anti-GAPDH and anti-D8 antibodies were used. **(D)** Immunostaining of MVA- or MVA-prME-infected HeLa cells (MOI 0.1, 24 hpi) stained against VACV D8 protein or TBEV E protein. Cells were non-permeabilized (surface) or treated with Triton X^®^-100 (intracellular). Images were taken with ×20 objective.**(E)** Growth curves of MVA- (gray) or MVA-prME (black)-infected permissive primary CEF (dotted lines) or non-permissive HeLa (solid lines) cells (MOI 0.05).

MVA-prME was generated by homologous recombination within deletion site III between MVA and a MVA transfer vector plasmid. To this end, sequences of C (only signal peptide), prM, and E based on the nucleotide sequence of TBEV Neudoerfl (UniProtKB: P14336) were *in silico* modified by the introduction of a silent mutation to avoid a repeated guanine sequence and by adding the Kozak sequence prior to the signal peptide of C. The gene sequence was synthesized (GenScript Biotech Corp, Piscataway Township, New Jersey, USA) and cloned into the MVA transfer vector plasmid pIIIsynIIred under transcriptional control of the VACV late promoter psynII (35) to obtain the recombinant vector plasmid (pIIIsynIIred-TBEV prME). pIIIsynIIred encompasses mCherry as a marker gene flanked by repetitive regions to allow deletion of mCherry by intragenomic homologous recombination. Recombinant MVA containing the synthesized TBEV sequence was generated using a modified standard protocol (35) (**Figure 1A**). MVA-prME was propagated on primary CEF cells. The virus stock was concentrated by ultracentrifugation at 38,400 rcf using 36% sucrose, and the virus preparation was resolved in tris-buffered saline (TBS, 120 mM NaCl/10 mM Tris–HCl, pH 7.4).

### 2.3 Determination of MVA-prME and MVA titers

Titration of MVA-prME and MVA was performed on primary CEF cells by MVA-specific immune peroxidase staining after a slight modification of the standard protocol (35) with overlay consisting of 1.25% Avicel, 1× MEM, 2% FBS, 1% Pen/Strep, and 1% MEM NEAA. Spots were counted, duplicates were averaged, and the virus titer was calculated to plaque-forming units (PFU) per ml. Virus titers are expressed as log_10_ PFU per ml.

### 2.4 *In vitro* characterization of MVA-prME

Integration of prME gene sequence into deletion site III of the MVA genome was demonstrated by PCRs targeting the six major deletion sites after a slight modification of the standard protocol (35) using GoTaq^®^ DNA polymerase (Promega, Madison, Wisconsin, USA). PCR products were purified with GeneJET Gel Extraction Kit (Thermo Scientific™, Waltham, Massachusetts, USA), DNA was separated on 1% agarose TBE gel and analyzed (ChemiDoc, ImageLab v6.0.1, Bio-Rad Laboratories GmbH, Feldkirchen, Germany). For control, non-recombinant MVA was used. Correct integration of the prME gene was verified by sequencing (Microsynth AG, Balgach, Switzerland) of purified DNA amplified by PCR targeting MVA-specific deletion site III (35). Expression of TBEV E protein was demonstrated in infected HeLa cells. To this end, cells were inoculated for 2 h at 37°C/5% CO_2_ with MVA-prME or MVA [multiplicity of infection (MOI) 5] or left untreated. The inoculum was replaced by the infection medium, and cells were harvested after 24 h in radioimmunoprecipitation assay buffer containing 1% Halt™ Protease-Inhibitor-Cocktail (100×) (Thermo Fisher Scientific, Waltham, Massachusetts, USA). Total protein concentration was measured using Pierce™ Detergent Compatible Bradford Assay Kit (Thermo Fisher Scientific, Waltham, Massachusetts, USA) with albumin standard (Thermo Scientific™, Waltham, Massachusetts, USA). Eight micrograms of total protein were separated under denaturing conditions on 10% Mini-PROTEAN^®^ TGX Stain-Free™ Protein Gels (Bio-Rad Laboratories GmbH, Feldkirchen, Germany) and blotted on Cytiva Amersham™ Hybond™ P 0.45 μm PVDF Membrane (VWR International GmbH, Darmstadt, Germany). Polyclonal rabbit Cell Surface-Binding Protein (D8L) Antibody (1:2,000, BIOZOL Diagnostica Vertrieb GmbH, Eching, Germany), mouse monoclonal TBEV E protein antibody (clone 19/1786, 1:500, kindly provided by Matthias Niedrig), GAPDH (D16H11) XP^®^ Rabbit mAb #5174 (1:3,000, Cell Signaling Technology^®, Danvers, Massachusetts, USA^), goat anti-rabbit IgG (H+L) HRP (1:5,000, Invitrogen, Waltham, Massachusetts, USA), and goat anti-mouse IgG (H+L) HRP (1:5,000, Invitrogen, Waltham, Massachusetts, USA) were used. Western blots were developed using SuperSignal™ West Pico PLUS Chemiluminescent Substrate (Thermo Scientific™, Waltham, Massachusetts, USA) and ChemiDoc Imaging System (ImageLab v6.0.1, Bio-Rad Laboratories GmbH, Feldkirchen, Germany). For immunostaining, HeLa cells were either infected with MVA-prME or MVA (MOI 1) or left uninfected. After 24 h, cells were fixed with 4% paraformaldehyde (Carl Roth GmbH + Co. KG, Karlsruhe, Germany), washed with 1× PBS, and either treated with 0.5% Triton X^®^-100 (Carl Roth GmbH + Co. KG, Karlsruhe, Germany) or left untreated. Antibodies for immunostaining were the same as for Western blot. Immunostaining was developed using TrueBlue™ Peroxidase Substrate (SeraCare, Milford, Massachusetts, USA). Images were taken with Leica DM IL LED (Leica LAS V4.5). Replication deficiency of MVA-prME and MVA was demonstrated on primary CEF and HeLa cells. Both cell lines were inoculated with the respective virus (MOI 0.05) for 1 h at 37°C/5% CO_2_. The inoculum was replaced by the infection medium, and samples were harvested after 0, 4, 12, 48, and 72 h post-infection (hpi). Viral titers were determined as described above.

### 2.5 Ethical statement

All mice experiments were performed in strict accordance with the European guidelines (EU directive on animal testing 2010/63/EU) and German Animal Welfare Law. The animal study was approved by the Lower Saxony State Office for Consumer Protection and Food Safety (approval no. 33.8-42502-04-20/3437).

### 2.6 Mice

Female C57BL/6JOlaHsd (C57BL/6) mice were purchased from Envigo RMS GmbH, Venray, Netherlands. Mice were housed under pathogen-free conditions in individually ventilated cages type Sealsafe Plus GM500 or IsoCage N Biocontainment system (Tecniplast, Hohenpeißenberg, Germany). Sterilized water and food were provided *ad libitum*. The experiments were started after 7 days of acclimatization of the mice. Treatment of the mice was always done under isoflurane anesthesia.

### 2.7 Immunogenicity study

C57BL/6 mice (6-8 weeks old, *n* = 4) were vaccinated intramuscularly (i.m.) with 10^7^ PFU MVA-prME (50 µl). Control mice were either vaccinated i.m. with TBS (50 µl), i.m. with 10^7^ PFU MVA (50 µl), or subcutaneously (s.c.) with 0.816 µg FSME-IMMUN^®^ (170 µl; Pfizer Pharma GmbH, Berlin, Germany, charge: EM2898). To minimize the number of experimental animals and to comply with the 3R principle (replacement, reduction, and refinement), data from MVA-vaccinated mice (empty vector control group) were shared with an experiment performed in parallel under identical experimental conditions (the same approval number). This was deemed justified because many studies failed to demonstrate any effect of the MVA vector control-induced immunity on immune responses to the pathogen of interest and protective efficacy [e.g (36–40)]. After 28 days, boost vaccination was performed. Fifty-six days after the first immunization, serum was obtained by puncturing the retrobulbar sinus (MiniCollect^®^ CAT Serum Sep Clot Activator tubes, Greiner Bio-One GmbH, Kremsmünster, Austria). Subsequently, mice were euthanized by cervical dislocation and spleens were harvested. Spleens were homogenized using gentleMACS™ Octo Dissociator (Miltenyi Biotec B.V. & Co. KG, Bergisch Gladbach, Germany) and cell strainers (Miltenyi Biotec B.V. & Co. KG, Bergisch Gladbach, Germany). Single-cell suspensions were treated with ACK Lysing buffer (Gibco™, Waltham, Massachusetts, USA) and resuspended in RPMI 1640 (1×) (Gibco™, Waltham, Massachusetts, USA) supplemented with 10% FBS, 1% Pen/Strep, and 5 mM ß-mercaptoethanol (R10F). During the immunization period, mice were monitored weekly for clinical signs according to the clinical score sheet including the categories "outer appearance", "behavior", "movement", "body weight", and "neurological signs".

### 2.8 Protective efficacy study

C57BL/6 mice (6-8 weeks old, *n* = 12) were vaccinated twice with MVA-prME, MVA, PBS (s.c., 100 µl), or FSME-IMMUN^®^ (Pfizer Pharma GmbH, Berlin, Germany, charge: EM2898) as described above. Health status was monitored weekly according to the clinical score sheet. Prior to prime immunization (d0), boost immunization (d28), and challenge infection (d56), blood was collected by puncture of the *Vena facialis*. Fifty-six days after prime immunization, mice were inoculated s.c. with 5.4 • 10^3^ tissue culture infectious dose 50% (TCID_50_) TBEV Neudoerfl (100 µl). Half of the group (*n* = 6) was taken out of the experiment after 8 days post-inoculation (dpi), whereas the remaining mice (*n* = 6) were kept until the study endpoint (16 dpi) or when the defined humane endpoint (HEP) was reached. Infected mice were scored daily. On the day of sacrifice, blood was collected by puncture of the retrobulbar sinus, and mice were euthanized by cervical dislocation. The organs were collected either for analysis of viral loads in PBS with a metal bead (the left brain hemisphere, cervical part of the spinal cord, spleen, rice-corn size of the ileum, rice-corn size of the colon) or for histopathological examination in ROTI^®^Histofix 4% (4% formaldehyde, Carl Roth GmbH + Co. KG, Karlsruhe, Germany, at least for 48 h) [the right brain hemisphere, the remaining gastrointestinal tract (GIT)]. The organs for the analysis of viral loads were homogenized using TissueLyser II (Qiagen, Hilden, Germany) at 30 Hz for 1 min.

### 2.9 Restimulation of spleen cells

15-mer peptides overlapped by 11 amino acids spanning the whole TBEV Neudoerfl E protein (UniProtKB: P14336) were synthesized (≥75% purity, GenScript Biotech Corp, Piscataway Township, New Jersey, USA). Lyophilized peptides were resolved in DMSO (Hybri-Max™, Sigma-Aldrich, St. Louis, Missouri, USA), and two peptide pools with 10 µg/ml of each peptide were generated (E_1-255_: 61 peptides, E_245-496_: 60 peptides). For *ex vivo* restimulation, spleen cells were incubated overnight at 37°C/5% CO_2_ with 1 µg/ml of the respective peptide pool, live MVA (MOI 3), DMSO/R10F (negative control), or a mixture of 30 ng/ml of phorbol 12-myristate 13-acetate (PMA; Cayman Chemical, Ann Arbor, Michigan, USA) and 0.5 µg/ml of ionomycin (Cayman Chemical, Ann Arbor, Michigan, USA) (positive control).

### 2.10 IFN-γ ELISpot assay

Restimulated splenocytes (2.5 • 10^5^/5 • 10^5^ cells/well; for positive control: 5 • 10^4^ cells/well) were tested for the frequency of IFN-γ-producing cells using IFN-γ ELISpot Plus kit (Mabtech, Nacka Strand, Sweden) according to the manufacturer’s instructions. Plates were scanned and analyzed with ImmunoSpot^®^ S6 Ultimate Reader (Cellular Technology Limited, Cleveland, Ohio, USA) and ImmunoSpot^®^ software (version 7.0.20.1, Cellular Technology Limited, Cleveland, Ohio, USA). Triplicates were averaged, background (DMSO/R10F) was subtracted, and the frequency of IFN-γ spot-forming cells (SFC) was expressed per 10^6^ splenocytes.

### 2.11 Flow cytometry

Restimulated splenocytes (10^6^ cells/well) were treated with 10 µg/ml of brefeldin A (Sigma-Aldrich, St. Louis, Missouri, USA) at 37°C/5%CO_2_ 4 h before staining. Spleen cells were stained with LIVE/DEAD™ Fixable Near-IR Dead Cell Stain Kit for 633 or 635 nm excitation (1:1,000, Invitrogen™, Waltham, Massachusetts, USA) followed by blocking of Fc receptors with CD16/CD32 Rat anti-Mouse (clone: 93, 1:500). For surface staining, antibodies to CD3e (clone: 145-2C11)-FITC, CD4 (clone: RM4-5)-PE, CD8a (clone: 53-6.7)-PerCP-Cyanine5.5, and CD69 (clone H1.2F3)-Alexa Fluor^®^ 700 (BD Biosciences, Franklin Lakes, New Jersey, USA) were used. Afterward, cells were fixed and permeabilized using BD Cytofix/Cytoperm™ (BD BioSciences, Franklin Lakes, New Jersey, USA). Intracellular staining was performed with antibodies to IFN-γ (clone: XMG1.2)-APC and Granzyme B (clone: QA18A28, BioLegend^®, San Diego, California, USA^)-BV421. Cells were suspended in PBS and acquired using BD LSR Fortessa X-20 and BD FACSDiva (version 9.0, BD Biosciences, Franklin Lakes, New Jersey, USA). Data were analyzed with FlowJo™ (version 10.8.1, BD Biosciences, Franklin Lakes, New Jersey, USA). Antibodies were used at 1:200 dilution and were purchased from eBioscience™ (Invitrogen™ Waltham, Massachusetts, USA) unless otherwise stated.

### 2.12 Virus neutralization assay

Heat-inactivated sera (56°C/30 min) of immunized mice were two-fold serially diluted in infection medium and mixed with 100 TCID_50_ TBEV Neudoerfl. After incubation for 1 h at 37°C/5% CO_2_, the serum–virus mix was transferred to A549 cells and incubated for 5-6 days at 37°C/5% CO_2_. Based on the occurrence of cytopathic effect (CPE), virus-neutralizing titer (VNT_100_), defined as the reciprocal of the highest serum dilution without detectable CPE, was determined.

### 2.13 Luciferase Immunoprecipitation System assay

The Luciferase Immunoprecipitation System (LIPS) assay targeting domain III of TBEV E protein was performed with heat-inactivated sera (56°C/30 min) as described previously (41). LIPS plasmids were kindly provided by Imke Steffen (Institute for Biochemistry and Research Center for Emerging Infections and Zoonoses, University of Veterinary Medicine Hannover, Foundation, Hannover, Germany). Triplicates were averaged and data were expressed as log_10_ relative light units (RLU), whereby values higher than the mean of naïve serum plus five times its standard deviation were considered positive. For one FSME-IMMUN^®^-vaccinated mouse, no serum was available on day 0.

### 2.14 Tissue culture infectious dose 50%

The sera and supernatants of cleared organ homogenates were 10-fold serially diluted in A549 infection medium and added to A549 cells. Cells were incubated at 37°C/5% CO_2_ and, after 5-6 days, screened for the presence/absence of CPE. TCID_50_ was determined according to the method of Reed and Muench (42), and infectious viral titers are expressed as log_10_ TCID_50_ per ml or gram tissue, respectively. The detection limit for each organ titration was calculated by dividing the lowest dilution (10^1^) by the respective averaged organ weight.

### 2.15 RNA isolation and real-time reverse transcriptase-quantitative PCR

The total RNA of supernatants of cleared organ homogenates was isolated using QIAmp^®^ Viral RNA Mini Kit (Qiagen, Hilden, Germany) according to the manufacturer’s instructions. Real-time reverse transcriptase-quantitative PCR (RT-qPCR) was performed using Luna^®^ Universal One-Step RT-qPCR Kit (New England BioLabs® GmbH, Frankfurt (Main), Germany) based on the modified protocol by Schwaiger and Cassinotti (43). For the quantification of TBEV RNA copies, a TBEV RNA standard (kindly provided by Stefanie Becker, Institute for Parasitology and Research Center for Emerging Infections and Zoonoses, University of Veterinary Medicine Hannover, Foundation, Hannover, Germany) was used. Nuclease-free water served as a negative control. Real-time RT-qPCR was performed with AriaMx Real-time PCR System with Agilent Aria software (version 1.5, Agilent Technologies, Santa Clara, California, USA). For data evaluation, quantification cycle (Cq) values of duplicates were averaged, and TBEV copies per gram of tissue were calculated. Data were expressed as log_10_ TBEV copies per gram tissue. In samples with no detectable viral RNA (no Cq value could be measured), calculation to log_10_ resulted in 1 (10^0^) copy per gram of tissue.

### 2.16 Histology and histological evaluation

Formaldehyde-fixed (ROTI^®^Histofix 4%, Carl Roth GmbH + Co. KG, Karlsruhe, Germany) tissue was processed by embedding two longitudinal sections of the brain and representative sections of the duodenum, jejunum, ileum, cecum, colon, and rectum in paraffin wax. The embedded tissue was cut into 2-3 µm thick sections using a microtome and stained with hematoxylin and eosin (H&E). For histological analysis, three brain regions (olfactory bulb, cerebral cortex, hippocampus) and six intestinal regions (duodenum, jejunum, ileum, cecum, colon, and rectum) were evaluated. In the brain, special emphasis was paid to cellular necrosis, perivascular parenchymal as well as vascular inflammation, microgliosis characterized by hyperplasia and/or hypertrophy of microglia/macrophages, and vascular lesions such as edema, hemorrhage, and fibrinoid necrosis. Intestinal regions were evaluated with special attention on inflammatory changes and/or signs of cellular, specifically neuronal necrosis within the *submucosal* as well as *myenteric plexus*.

### 2.17 Immunohistochemistry and immunohistochemical evaluation

For immunohistochemistry (IHC), the avidin–biotin–peroxidase complex (ABC) method using a mouse monoclonal TBEV E protein-specific antibody (clone 19/1493, 1:2,000, kindly provided by Matthias Niedrig) was performed as described previously (44, 45). 3,3′-diaminobenzidine tetrahydrochloride (DAB) served as a chromogen, and nuclei were counterstained with Mayer’s hematoxylin (Carl Roth GmbH + Co. KG, Karlsruhe, Germany). The olfactory bulb, cerebral cortex, and hippocampus were examined with respect to TBEV-positive cells [single immunopositive cells: 1-5 cells per high power field (HPF); low numbers of immunopositive cells: 6-10 cells per HPF; moderate numbers of immunopositive cells: 11-15 cells per HPF; high numbers of immunopositive cells: >15 cells per HPF]. The distribution of TBEV immunoreaction within the brain regions was evaluated as either focal, multifocal, or diffuse. In the intestine, TBEV-positive cells located in the *submucosal* and *myenteric plexus* were evaluated analogously.

### 2.18 Statistics

For statistical analysis, GraphPad Prism software (version 9.0.0, GraphPad Software Inc., Boston, Massachusetts, USA) was used. For all statistical tests, the unpaired *t*-test was used. For survival data, Kaplan–Meier curves and log-rank test were used. A *p*-value <0.05 was considered significant.

## 3 Results

### 3.1 Recombinant MVA expressing prME of TBEV

Successful integration of the TBEV prM and E genes in deletion site III of MVA (**Figure 1A**) was confirmed by specific PCRs targeting the six major deletion sites of MVA (**Figure 1B**). Nucleotide sequencing of prME confirmed the completeness and absence of mutations (data not shown). Protein expression of TBEV E was confirmed by Western blot using lysates of HeLa cells harvested 24 hpi (**Figure 1C**). In addition, immunostaining of MVA-prME-infected and permeabilized HeLa cells using a TBEV E-specific antibody confirmed the expression of the E protein. The E protein was not detected in non-permeabilized cells, indicating that it was absent on the host cell surface (**Figure 1D**). Furthermore, the expression of prME did not affect the replication deficiency of MVA in mammalian cells. MVA-prME and MVA replicated to high titers in primary CEF cells, whereas both viruses displayed restricted replicative capacity in human HeLa cells (**Figure 1E**).

Thus, *in vitro* characterization of MVA-prME confirmed the integration of the prME gene sequence in deletion site III of the MVA genome, the synthesis of TBEV E protein in infected cells, and the attenuated phenotype of MVA.

### 3.2 MVA-prME is well tolerated and immunogenic in mice

Mice were immunized twice 4 weeks apart with 10^7^ PFU MVA-prME. As controls, mice were either vaccinated with TBS, 10^7^ PFU MVA (empty vector control), or 0.816 µg FSME-IMMUN^®^. The health status of mice was monitored weekly over a period of 56 days. All mice showed increasing body weights and did not show any clinical signs upon immunization (**Supplementary Figure S1**).

**Figure 2:**
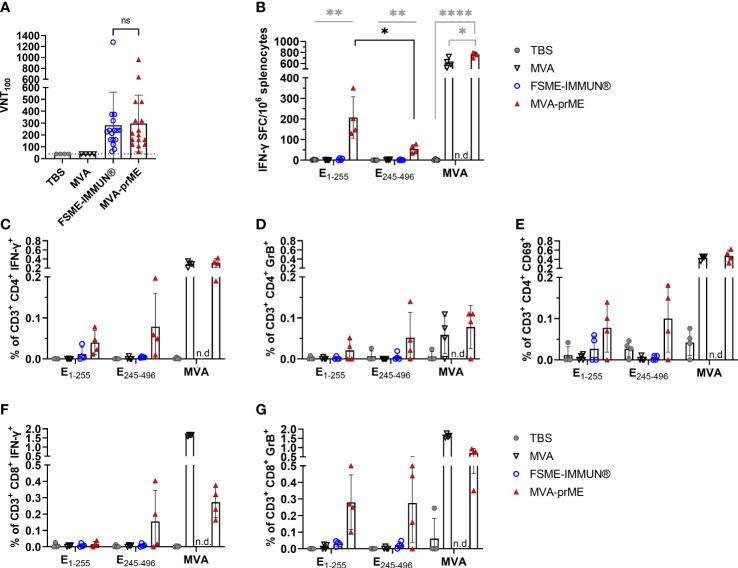
Humoral and cellular immune response in vaccinated mice. **(A)** Virus-neutralizing titer (VNT_100_) against TBEV Neudoerfl of murine sera samples obtained 56 days after prime immunization (samples of immunogenicity and protective efficacy study, *n* = 16). Samples with ≤40 VNT_100_ (dotted line, lowest serum dilution) are considered negative. The FSME-IMMUN^®^-vaccinated mouse that displayed signs of disease is highlighted with a diamond symbol. n.s., not significant (*p*>0.05). **(B)** Displayed are IFN-γ spot-forming cells (SFC) per one million splenocytes after background subtraction. Only significance between MVA-prME to other treatment groups is indicated (gray) and for E_1-255_ *versus* E_245-496_ of MVA-prME-vaccinated mice (black) (*p≤0.05, **p≤0.01, ****p≤0.0001). **(C–G)** Frequency of CD3^+^ subpopulations gated on CD4^+^IFN-γ^+^ **(C)**, CD4^+^Granzyme B^+^ **(D)**, CD4^+^CD69^+^ **(E)**, CD8^+^IFN-γ^+^ **(F)**, or CD8^+^Granzyme B^+^ **(G)** after background substraction. n.d., not determined. For all graphs, bars show the mean with standard deviation. Mice were either immunized with TBS (gray circle), MVA (non-filled triangle), FSME-IMMUN^®^ (non-filled blue circle), or MVA-prME (red triangle).

The serum obtained 56 days after the first immunization was used for the TBEV neutralization assay (**Figure 2A**). Administration of TBS or empty MVA did not result in the induction of TBEV*-*specific antibodies. In contrast, the sera of all mice vaccinated with MVA-prME or FSME-IMMUN^®^ displayed neutralizing activity against TBEV. Mean VN titers after MVA-prME (296 VNT_100_) or FSME-IMMUN^®^ (280 VNT_100_) vaccination did not significantly differ.

Furthermore, we examined the T cell response against the TBEV E protein and MVA by *ex vivo* restimulation of splenocytes obtained 56 days after the first immunization. MVA-prME-vaccinated mice displayed specific T cells responding to both peptide pools E_1-255_ and E_245-496_, as detected by IFN-γ ELISpot assay (**Figure 2B**), which were not detected in the FSME-IMMUN^®^-vaccinated mice and the control groups. The response to the E_1-255_ peptide pool was significantly higher than that to the E_245-496_ pool. In addition, in mice that received MVA-prME or MVA vector control, a strong MVA-specific T cell response was observed (**Figure 2B**). To further characterize the cellular response, flow cytometry was performed on splenocytes after *ex vivo* restimulation. In MVA-prME-immunized mice, specific CD4^+^ T cells were the main source of IFN-γ in response to the E_1-255_ peptide pool and MVA restimulation (**Figure 2C**). In response to the E_245-496_ peptide pool, IFN-γ was induced in both specific CD4^+^ and CD8^+^ T cells (**Figures 2C, F**). In addition, Granzyme B in CD4^+^ and predominantly CD8^+^ T cells was detected against both peptide pools in MVA-prME-vaccinated mice (**Figures 2D, G**). After stimulation with both peptide pools, the expression of the early activation marker CD69 was only detected in CD4^+^ T cells in three out of four MVA-prME-vaccinated mice (**Figure 2E**). Within the group of MVA-prME-vaccinated mice, the frequency of TBEV E-specific T cells was variable, whereas the frequency of MVA-specific T cells was relatively homogeneous.

Taken together, we showed that MVA-prME is well tolerated in mice and is capable of inducing TBEV-specific VN antibodies as well as TBEV E protein- and MVA-specific CD4^+^ and CD8^+^ T cells. In contrast, immunization with FSME-IMMUN^®^ resulted in the induction of VN antibodies, but T cell responses were not detectable.

### 3.3 MVA-prME fully protects mice against TBEV challenge infection

To examine whether MVA-prME vaccination afforded protection against TBEV challenge infection, mice were immunized twice with MVA-prME, PBS, empty MVA vector control, or FSME-IMMUN^®^ and subsequently inoculated with a lethal dose of TBEV Neudoerfl. The induction of VN antibodies in MVA-prME- and FSME-IMMUN^®^-vaccinated mice before challenge infection was confirmed (**Figure 2A**) and coincided with the development of antibodies directed to domain III (DIII) of the TBEV E protein, as measured by LIPS assay (**Supplementary Figure S2B**). Furthermore, LIPS assay showed an increase in serum antibody levels to DIII after each vaccination (**Supplementary Figures S2A, B**).

**Figure 3:**
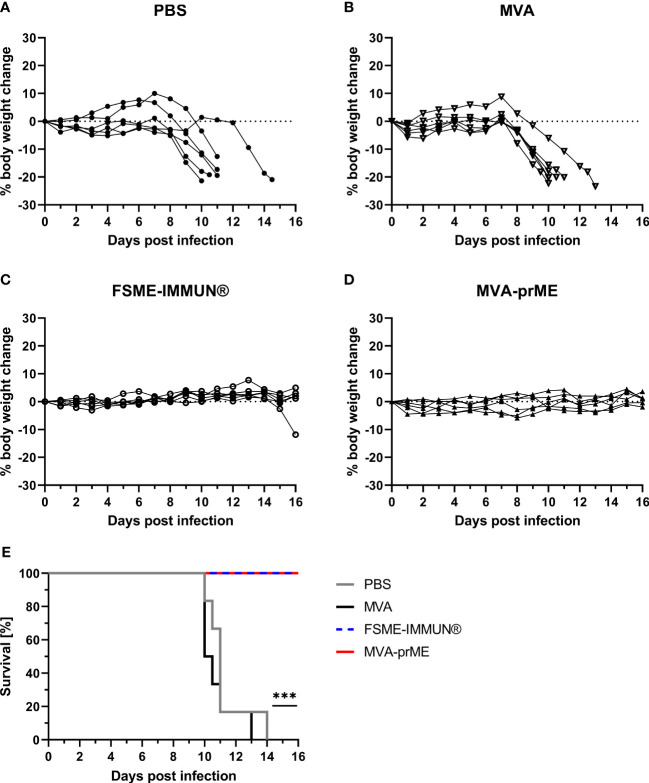
Body weight change and survival curves of vaccinated and challenge infected mice. **(A–D)** Percentage of body weight change compared with initial body weight on the day of challenge during the entire course of TBEV infection for PBS- **(A)**, MVA- **(B)**, FSME-IMMUN^®^- **(C)**, and MVA-prME-vaccinated **(D)** mice. **(E)** Kaplan–Meier curves showing the percentage of survival of mice vaccinated with PBS (gray), MVA (black), FSME-IMMUN^®^ (blue, dotted), or MVA-prME (red) (n = 6, ***p≤0.001). *p* = 0.0007: FSME-IMMUN^®^/MVA-prME *versus* PBS, *p* = 0.0004: FSME-IMMUN^®^/MVA-prME *versus* MVA.

All PBS and MVA control mice developed clinical signs and displayed weight loss following infection with TBEV Neudoerfl starting at 7-8 dpi (**Figures 3A, B**). Clinical signs included abnormal outer appearance (e.g., hunched back, dull fur) and reduced spontaneous and induced activity as well as walking on tiptoes. Furthermore, two out of six mice in the PBS group showed signs of neurological disease. These mice reached the predefined HEP between 10 and 14 dpi and had to be taken out of the experiment (**Figure 3E**). In contrast, mice vaccinated with MVA-prME or FSME-IMMUN^®^ did not display weight loss or clinical signs of infection (**Figures 3C, D**). However, one mouse in the FSME-IMMUN^®^ group started to lose body weight at 14 dpi onward and displayed abnormal movement by walking on tips. All MVA-prME- and FSME-IMMUN^®^-vaccinated mice survived until the study endpoint (**Figure 3E**).

#### 3.3.1 Vaccination with MVA-prME reduces viral load in the spleen, central nervous system, and gastrointestinal tract

To assess virus replication, viral loads in the serum and organs were determined on the day of sacrifice (**Supplementary Figure S3**). In PBS- and MVA-vaccinated mice, high infectious virus titers were detected in the tissues from the central nervous system (CNS) and colon at 8 dpi. In the serum, spleen, and ileum, no infectious virus was detected (**Supplementary Figure S3A**). None of the mice in the MVA-prME group had infectious virus in the serum and tissue samples collected at 8 and 16 dpi (**Supplementary Figures S3A, B**). Similar results were observed for FSME-IMMUN^®^-vaccinated mice (**Supplementary Figures S3A, B**). However, one mouse of this group sacrificed on 16 dpi had high infectious TBEV titers in the brain (10^8^ TCID_50_/gram tissue) and spinal cord (10^9^ TCID_50_/gram tissue) (**Supplementary Figure S3B**). Of note, it was the same mouse that lost body weight and developed clinical signs upon TBEV challenge infection.

**Figure 4:**
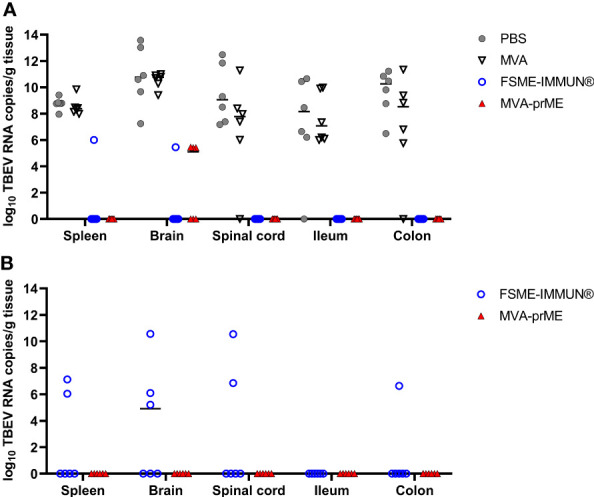
Quantification of TBEV RNA copies in the periphery, CNS, and GIT of vaccinated and infected mice. Cleared organ homogenates collected on the day of sacrifice were tested for the presence of TBEV RNA by real-time RT-qPCR. Mice were either sacrificed at **(A)** 8 dpi or **(B)** stayed in the experiment until the study endpoint (16 dpi). The median values of the respective treatment groups are indicated in the graphs. Mice were immunized with PBS (gray circle), MVA (non-filled triangle), FSME-IMMUN^®^ (non-filled blue circle), or MVA-prME (red triangle).

Furthermore, tissue samples were analyzed by real-time RT-qPCR (**Figure 4**). Mice from the PBS and MVA groups euthanized at 8 dpi had high TBEV RNA copy numbers in the spleen (10^7^-10^9^ RNA copies/gram tissue), CNS (brain: 10^7^-10^13^ RNA copies/gram tissue, spinal cord: 10^6^-10^12^ RNA copies/gram tissue), and GIT (ileum: 10^6^-10^10^ RNA copies/gram tissue, colon: 10^5^-10^11^ RNA copies/gram tissue) (**Figure 4A**). One mouse in the PBS group did not display viral RNA in the ileum, and in two MVA-vaccinated mice, viral RNA was not detected in either the spinal cord or colon. At 8 dpi, three out of six MVA-prME-vaccinated mice displayed low levels of TBEV RNA (10^5^ RNA copies/gram tissue) in the brain (**Figure 4A**). Two mice vaccinated with FSME-IMMUN^®^ showed viral RNA (10^5^ RNA copies/gram tissue) either in the spleen or brain (**Figure 4A**). Other organs of both treatment groups were RNA-negative. In most mice that developed VN antibodies upon vaccination, no viral RNA could be detected in the spleen, spinal cord, ileum, and colon at 8 dpi (**Supplementary Figure S4**).

Samples collected at 16 dpi were all negative for TBEV RNA in mice immunized with MVA-prME (**Figure 4B**). In contrast, viral RNA was detectable in the brain (3/6), spinal cord (2/6), spleen (2/6), and colon (1/6) samples of mice immunized with FSME-IMMUN^®^ (**Figure 4B**).

#### 3.3.2 Vaccination with MVA-prME prevents pathological alterations in the CNS and reduces GIT pathology

**Figure 5:**
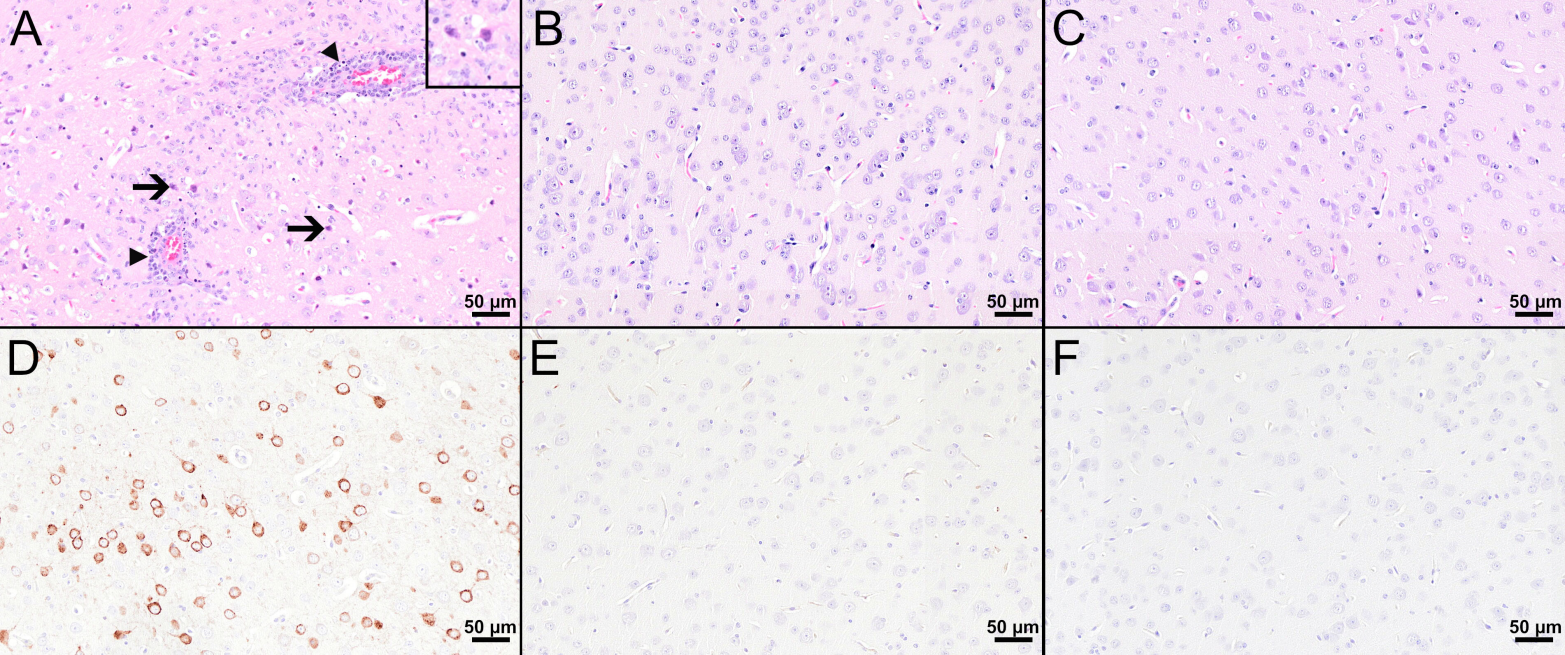
Histological and immunohistochemical analyses of the cerebral cortex at 8 dpi. **(A–C)** H&E-stained sections of the cerebral cortex of TBEV-infected mice which were either treated with PBS **(A)** or vaccinated with FSME-IMMUN^®^ **(B)** or MVA-prME **(C)**. **(A)** The cerebral cortex of the PBS-treated mouse displays cellular necrosis with karyorrhectic, karyolytic, and pyknotic cells (insert) and shrunken, hypereosinophilic, triangular-shaped necrotic neurons (arrows and insert) as well as inflammatory cell infiltrates in destructed vascular walls (necrotizing vasculitis; arrowheads) and the perivascular space (arrowheads). Microgliosis and hypertrophy of microglia/macrophages are present. **(B, C)** In FSME-IMMUN^®^- **(B)** or MVA-prME-vaccinated **(C)** mice, no significant microscopic lesions within the cerebral cortex parenchyma are visible. **(D–F)** IHC for the TBEV E antigen of the cerebral cortex of TBEV-infected mice which were either treated with PBS **(D)** or vaccinated with FSME-IMMUN^®^ **(E)** or MVA-prME **(F)**. **(D)** Immunohistochemically, a cytoplasmic TBEV immunoreactivity is present in multiple cells representing neurons of the cerebral cortex from a PBS-treated mouse. **(E, F)** The cerebral cortex of FSME-IMMUN^®^- **(E)** and MVA-prME-vaccinated **(F)** mice lack immunoreactivity. Scale bars: 50 µM.

**Figure 6:**
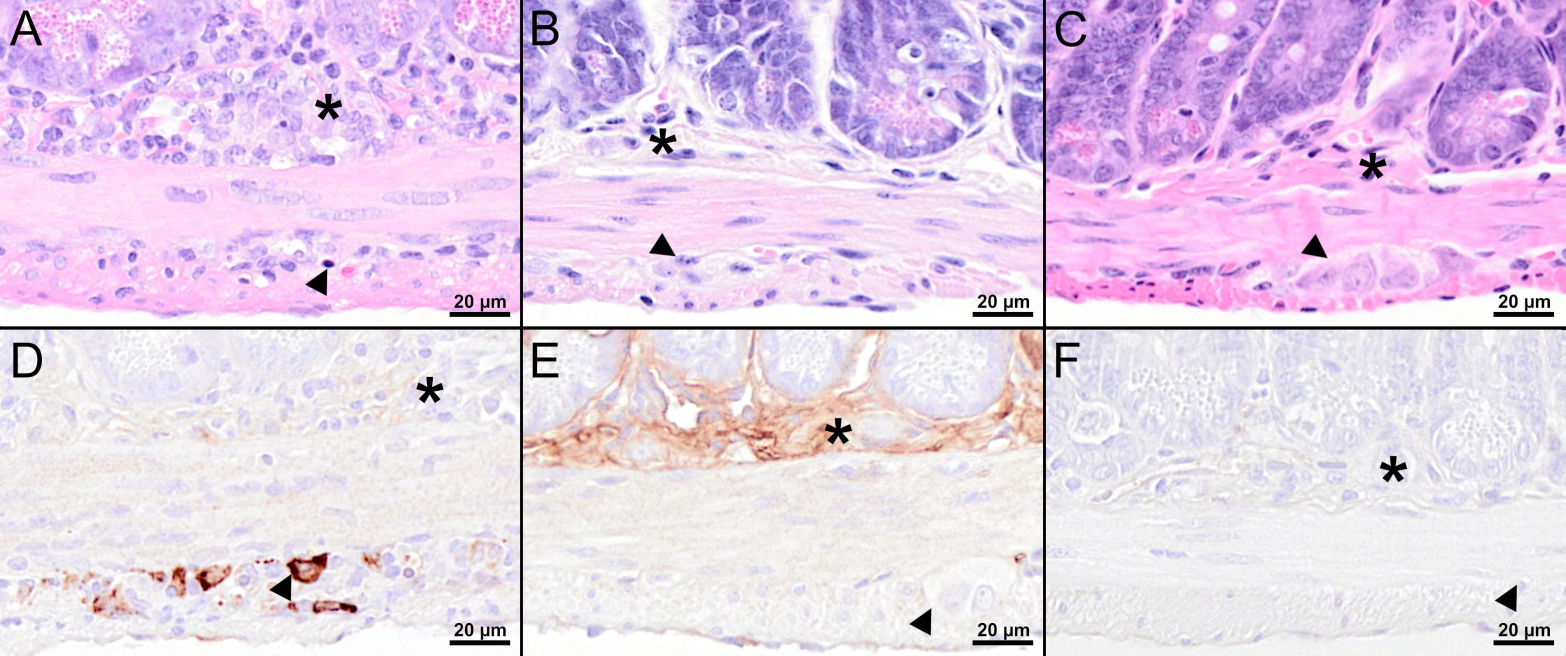
Histological and immunohistochemical analyses of the jejunum at 8 dpi. **(A–C)** H&E-stained sections of the jejunum of TBEV-infected mice which were either treated with PBS **(A)** or vaccinated with FSME-IMMUN^®^ **(B)** or MVA-prME **(C)**. **(A)** Marked hypercellularity/inflammatory cell infiltrates in the *submucosal plexus* (asterisk) and, to a lesser extent, in the *myenteric plexus* (arrowhead) as well as cellular necrosis with karyorrhectic, karyolytic, and pyknotic cells (arrowhead) are present. **(B, C)** No significant microscopic lesions within the *submucosal* (asterisk) and *myenteric plexus* (arrowhead) are present in vaccinated mice. **(D–F)** IHC for TBEV E antigen of the jejunum of TBEV-infected mice which were either treated with PBS **(D)** or vaccinated with FSME-IMMUN^®^ **(E)** or MVA-prME **(F)**. **(D)** Immunohistochemically, TBEV-positive neurons are observed in the *myenteric plexus* (arrowhead) but not in the *submucosal plexus* (asterisk). **(E, F)** No immunopositive cells are present in the *submucosal* (asterisk) or *myenteric plexus* (arrowhead). Band-like, not cell-associated, brownish discoloration in **(E)** represents a staining artifact due to the unspecific binding of the antibody to serum components. Scale bars: 20 µM.

Histopathological analysis of the mouse in the PBS group with the highest viral load in the CNS revealed marked histopathological lesions within the brain at 8 dpi (**Figure 5A**). All three brain regions (olfactory bulb, cerebral cortex, hippocampus) showed multifocal moderate to severe, mostly perivascular and vascular infiltrations with inflammatory cells as well as mild to moderate hypertrophy and hyperplasia of microglia/macrophages (microgliosis). Furthermore, multifocally variable numbers of necrotic cells were observed (**Figure 5A**). IHC for TBEV of the brain revealed high numbers of immunopositive cells, representing neurons (**Figure 5D**). One mouse in the PBS group had high virus burden in the GIT at 8 dpi. Histopathologically, the duodenum, jejunum, ileum, and colon of this mouse showed moderate to severe lesions characterized by hypercellularity/inflammatory cell infiltration as well as signs of cellular necrosis in the *submucosal* and *myenteric plexus* (**Figure 6A**). In addition, the cecum and rectum of this mouse showed the abovementioned histopathological abnormalities, although alterations were only minimal to mild. Accordingly, low to high numbers of TBEV-immunopositive cells, representing neurons, were found in both plexuses (**Figure 6D**).

**Figure 7:**
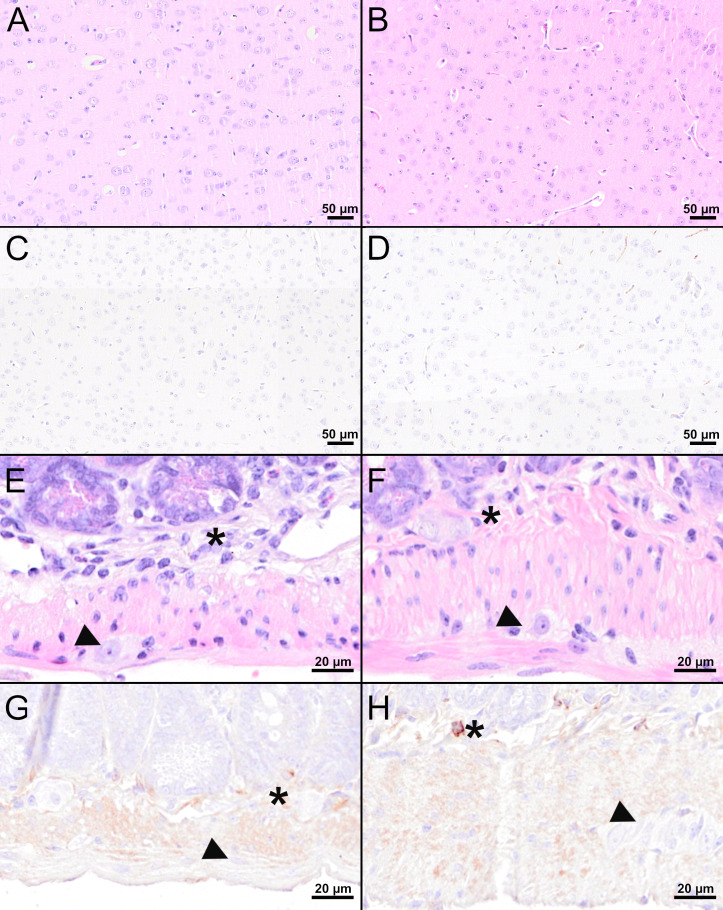
Histological and immunohistochemical analyses of the cerebral cortex and jejunum at 16 dpi. **(A, B, E, F)** H&E-stained sections of the cerebral cortex **(A, B)** and jejunum **(E, F)** of TBEV-infected mice which were either vaccinated with FSME-IMMUN^®^ **(A, E)** or MVA-prME **(B, F)**. **(C, D, G, H)** IHC for TBEV E antigen of the cerebral cortex **(C, D)** and jejunum **(G, H)** of TBEV-infected mice either vaccinated with FSME-IMMUN^®^ **(C, G)** or MVA-prME **(D, H)**. **(A, B)** No significant microscopic lesions within the cerebral cortex parenchyma are visible. **(C, D)** TBEV immunoreactivity was absent within the parenchyma of the cerebral cortex. **(E, F)** The jejunum shows mild hypercellularity of the *submucosal plexus* (asterisk), while the *myenteric plexus* (arrowhead) reveals no significant findings. **(G)** No specific TBEV immunoreactivity is detectable in the *submucosal* (asterisk) or the *myenteric plexus* (arrowhead). **(H)** A single cell in the *submucosal plexus* (asterisk) is immunolabeled, while no immunoreactivity is present in the *myenteric plexus* (arrowhead). Scale bars: **(A–D)** 50 µM, **(E–H)**: 20 µM.

Despite detectable RNA in mice vaccinated with MVA-prME or FSME-IMMUN^®^, histopathological evaluation of the brain from representative mice was without significant microscopic lesions (**Figures 5B, C**). In addition, no TBEV-positive cells were found in the cerebral cortex of these mice (**Figures 5E, F**). Moreover, the GIT of representative MVA-prME- and FSME-IMMUN^®^-vaccinated mice were mostly without significant histopathological lesions (**Figures 6B, C**). Few animals displayed minimal to mild inflammatory cell infiltrates in the *submucosal plexus* (data not shown). IHC evaluation showed that mice in both groups either had no TBEV-immunopositive cells or only single to low numbers of TBEV-positive cells within the *submucosal plexus* (**Figures 6E, F**). Even though no TBEV RNA was detectable in the samples collected at 16 dpi, these findings could not be completely confirmed by histological and immunohistochemical analyses (**Figure 7**). On the one hand, MVA-prME- and FSME-IMMUN^®^-vaccinated representative mice displayed no significant parenchymal lesions in the cerebral cortex (**Figures 7A, B**), and no TBEV immunoreactivity was seen within the brain (**Figures 7C, D**). On the other hand, in the intestine of these mice, the jejunum and cecum displayed mild to moderate inflammatory cell infiltrations in the *submucosal plexus* (**Figures 7E, F**). Furthermore, the ileum of an MVA-prME-vaccinated mouse showed mild to moderate inflammatory cell infiltrates in both plexuses (**Figure 7F**). Immunohistochemically, no or single to low numbers of TBEV-positive cells, mostly detectable in the neurons of the *submucosal plexus*, were seen in the intestine of these mice (**Figures 7G, H** **)**.

Thus, residual TBEV replication was observed in MVA-prME- and FSME-IMMUN^®^-vaccinated mice, but this was more prominent in mice immunized with FSME-IMMUN^®^, especially at 16 dpi. However, MVA-prME afforded full protection against lethal TBEV challenge infection. In contrast, one mouse in the FSME-IMMUN^®^ group displayed severe signs of infection.

## 4 Discussion

TBE is a vaccination-preventable serious disease in humans. Despite the availability of inactivated vaccines, the number of confirmed TBE cases is rising (2, 3), including patients that were fully vaccinated (15–20).

To our knowledge, this is the first study using MVA as a viral vector to deliver antigens of TBEV. We successfully inserted the prM and E genes of TBEV Neudoerfl in deletion site III of MVA (MVA-prME) and confirmed intracellular E protein expression in non-permissive human cells. Since blockade of the MVA replication cycle occurs late in mammalian cells, the protein biosynthesis of MVA and foreign genes is not affected (24). Further characterization confirmed that the insertion of the prM and E genes did not affect the known replication deficiency of MVA in human cells which contributes to its safety profile allowing its use in infants, adults, elderly, and immunocompromised patients (46–49).

For many flaviviruses, it was shown that the co-expression of prM and E results in the assembly of virus-like particles (VLPs) lacking the viral genome (50). TBEV-derived VLPs are structurally very similar to native virions and show the same immunogenic reactivity toward monoclonal antibodies (21, 51). TBEV-derived VLPs produced by co-expression of prM and E were previously demonstrated using various expression systems (51–53). Based on the results from previous studies, we assume that the use of MVA-prME also resulted in the production of VLPs.

Vaccination of C57BL/6 mice with MVA-prME and FSME-IMMUN^®^ was well tolerated as expected, and both vaccine preparations were immunogenic and induced similar high VN antibody titers. VN antibodies are an important correlate of protection against TBEV infection, and a VN serum antibody titer ≥10 is considered protective in humans. The seroconversion rate of mice vaccinated with MVA-prME and FSME-IMMUN^®^ was 100%. The flaviviral glycoprotein E consists of four domains (DI-IV) (54), of which DI-III are the main targets for antibodies (55). Sera obtained post-vaccination with MVA-prME or FSME-IMMUN^®^ displayed strong reactivity with TBEV DIII. Serum antibodies were boosted by a second dose of MVA-prME, indicating that the anti-vector response had no influence on the EDIII-specific antibody titers. This is supported by clinical data demonstrating that a late boost after 1 or 4 years increased the antigen-specific antibody response (29, 33, 56) but only moderately the T cell response (33, 56). Since only antibodies to DIII were tested, it cannot be excluded that the induction of antibodies to the other domains differed between MVA-prME and FSME-IMMUN^®^, which is an aluminum hydroxide-adjuvanted, formaldehyde-inactivated virus preparation.

For several flaviviruses, antibody-dependent enhancement (ADE) of infection due to pre-existing non-neutralizing antibodies or suboptimal concentrations of neutralizing antibodies has been reported (57). However, the potential risk of MVA-prME-induced antibodies to contribute to ADE of TBEV infection is unlikely because proof of *in vivo* ADE is lacking and so far ADE was only observed *in vitro* (58–60).

Also, T cells may play a role in the pathogenesis of TBEV infection. Both virus-specific CD4^+^ and CD8^+^ T cells can contribute to protective immunity against TBE, but CD8^+^ T cells may also exert detrimental effects (10). In the present study, a cell-mediated immune response could be demonstrated only in MVA-prME-immunized mice. The response was significantly stronger against the N-terminal part of the E protein (E_1-255_). Further deconvolution would allow identifying single immunodominant epitopes recognized by C57BL/6 mice which was beyond the scope of the present study. Using flow cytometry, it was confirmed that both CD4^+^ and CD8^+^ T cells contributed to the E protein-specific T cell response. These virus-specific T cells displayed cytolytic properties as evidenced by their production of Granzyme B. Expression of the early activation marker CD69 was observed in CD4^+^ T cells only. Several studies have demonstrated E-specific CD4^+^ T cell responses in humans receiving TBE vaccination (61–64) characterized by high IL-2 and TNF-α but low IFN-γ production (61, 62, 64). Recently, TBEV-specific CD8^+^ T cell responses were observed in individuals that received the Russian TBE vaccine Tick-E-Vac^®^ (65). Therefore, we cannot exclude that the mice that received FSME-IMMUN^®^ mounted a virus-specific T cell response, albeit below the detection limit. The fact that they developed a strong antibody response would support this possibility. The mice that received MVA-prME also developed CD4^+^ and CD8^+^ T cell responses to the vector.

Next, we tested the protective efficacy of the respective vaccine preparations against a lethal challenge infection with the homologous TBEV strain Neudoerfl and determined the clinical outcome of infection as well as virus replication in the periphery, CNS, and GIT and associated histopathological changes. In line with previous studies, all control animals displayed body weight loss starting at 7-8 dpi and succumbed to infection between 10 and 14 dpi (66, 67). After 8 dpi, TBEV already replicated to high copy numbers in the periphery, CNS, and GIT. Additionally, neuroinvasion and neurovirulence were confirmed histopathologically and immunohistochemically in the brain tissue sections of a representative unprotected control mouse. Moreover, morphological changes of the GIT including cellular necrosis and hypercellularity/inflammatory cell infiltration were observed in the *submucosal plexus* and, to a lesser extent, in the *myenteric plexus* of an unprotected control mouse. Enteric ganglioneuritis of the *submucosal* and *myenteric plexus* after TBEV infection was reported in mice previously (67). In contrast, all mice vaccinated with MVA-prME were 100% protected without the development of TBE-associated clinical signs. Interestingly, FSME-IMMUN^®^ failed to afford complete protection since one mouse in this group had lost 11% of her initial body weight at 16 dpi (the study endpoint). The probability to reach the predefined HEP one day later was very likely (66). Remarkably, the VN titer of this mouse prior to challenge infection was comparable with the VN titers of mice in the same group, indicating that this mouse had responded to the vaccine. It is unclear why this mouse displayed serious disease progression despite having a high VN titer.

Except for the one FSME-IMMUN^®^-vaccinated mouse, all MVA-prME- and FSME-IMMUN^®^-immunized mice were protected from TBEV infection of the CNS and GIT. However, as indicated by real-time RT-qPCR data, no sterilizing immunity upon MVA-prME and FSME-IMMUN^®^ vaccination was induced since TBEV RNA was detected in a number of spleen or brain samples at 8 dpi. Nevertheless, compared with control groups, median viral loads in the brain were around 10^5^-fold lower in these mice. In the organ samples of MVA-prME-vaccinated mice collected at 16 dpi, no viral RNA could be detected. In contrast, in FSME-IMMUN^®^-vaccinated mice, TBEV RNA was detected in the periphery, CNS, and GIT at this time point post-infection. We speculate that the induction of cell-mediated immunity in MVA-prME-vaccinated mice contributed to protective immunity which could explain the more accelerated clearance of the infection compared with FSME-IMMUN^®^-vaccinated mice. However, the absence of an infectious virus in both treatment groups suggests that TBEV infection was restricted to a large extent. Moreover, the brain and GIT of MVA-prME- and FSME-IMMUN^®^-vaccinated mice collected at 8 dpi were histopathologically and immunohistochemically without significant findings. However, few mice, except one mouse, displayed mild histopathological changes in some intestinal regions as well as single to low numbers of TBEV-positive cells. Our results are in concordance with previous studies showing that vaccination with FSME-IMMUN^®^ or the adoptive transfer of serum can protect mice against the development of clinical signs without affording sterile immunity (68, 69). Thus, high levels of serum antibodies that can neutralize *in vitro* do not always coincide with full protection against infection *in vivo* (68). In addition, it was shown that neuroinvasion of TBEV was found in mice recovering from TBE, indicating that the detection of TBEV RNA combined with histological and immunohistochemical findings in the brain does not per se correlate with the lethal outcome of the infection (66).

In MVA-prME- and FSME-IMMUN^®^-vaccinated mice, no infectious virus was detected in the serum and spleen at 8 and 16 dpi, which is in agreement with the fact that viremia only takes place during the first phase after contracting TBEV (66). The results of our study are in agreement with those of other studies, which showed that delivery of TBEV prME can induce VN antibodies and afford protection from infection in animal models (21, 52, 70, 71). The fact that mice in the FSME-IMMUN^®^ group were protected from infection in the absence of detectable virus-specific cell-mediated immune responses is in agreement with the previously reported observation that VN antibodies are an important correlate of protection (58, 68). Nevertheless, and as outlined above, the induction of virus-specific T cell response may well have contributed to protective immunity and accelerated clearance of infection as observed in the MVA-prME-vaccinated mice.

In summary, the combination of MVA as a viral vector and prME as vaccine target antigens showed to be a highly promising approach to vaccinate against TBEV infection. We showed that vaccination with MVA-prME is well tolerated in mice and induces strong TBEV-specific VN antibodies comparable to vaccination with the licensed vaccine FSME-IMMUN^®^. Furthermore, with MVA-prME, TBEV E protein-specific CD4^+^ and CD8^+^ T cell responses were induced, which were not observed after vaccination with FSME-IMMUN^®^ and which correlated with accelerated clearance of the infection. MVA-prME vaccination afforded full protection against lethal TBEV challenge infection. Considering these favorable results and the excellent safety profile of MVA-based vaccines, further evaluation and clinical testing of MVA-prME as a next-generation vaccine candidate against TBEV seems warranted.

## Data availability statement

The original contributions presented in the study are included in the article/**Supplementary Material**. Further inquiries can be directed to the corresponding author/s.

## Ethics statement

The animal study was reviewed and approved by the Lower Saxony State Office for Consumer Protection and Food Safety (LAVES, Approval No. 33.8-42502-04-20/3437).

## Author contributions

Conceptualization: JBe, MK, TG, CKP, and GR. Formal analysis: JBe and MK. JBe, MK, JBi, and CKP performed the animal experiments and analyzed the samples. IZ, CP, and WB performed and analyzed the histology and immunohistochemistry data. Resources: GS. Writing—original draft preparation: JBe and MK. Writing—review and editing: IZ, CP, JBi, TG, AT, WB, AO, GS, CKP, and GR. Visualization: JBe and MK. Supervision: TG, CKP, and GR. Funding acquisition: GR. All authors have read and agreed to the published version of the manuscript.
