# Supplementary material for: A recombinant Modified Vaccinia virus Ankara expressing prME of tick-borne encephalitis virus affords mice full protection against TBEV infection
Source: Front Immunol. 2023 Apr 21;14:1182963. doi: 10.3389/fimmu.2023.1182963 (PMC10160477; doi:10.3389/fimmu.2023.1182963)
Supplement: Supplementary file 1 [file DataSheet_1.docx]

Supplementary Material

A recombinant Modified Vaccinia virus Ankara expressing prME of tick-borne encephalitis virus affords mice full protection against TBEV infection

Mareike Kubinski^1^**^†^**, Jana Beicht^1^**^†^**, Isabel Zdora^2,3^, Jeannine Biermann^1^, Christina Puff^2^, Thomas Gerlach^1^, Alina Tscherne^4,5^, Wolfgang Baumgärtner^2,3^, Albert D. M. E. Osterhaus^1^, Gerd Sutter^4,5^, Chittappen Kandiyil Prajeeth^1^ and Guus F. Rimmelzwaan^1*^

*** Correspondence:** Guus F. Rimmelzwaan (Guus.Rimmelzwaan@tiho-hannover.de)

# Supplementary Figures


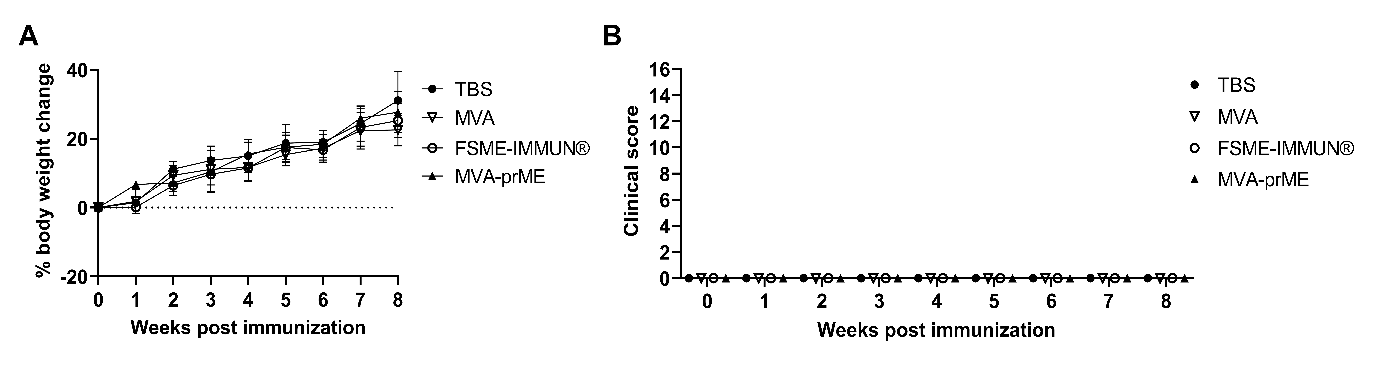


**Supplementary Figure 1.** Tolerability of MVA-prME in mice. (A) Percentage of body weight change and (B) clinical score of vaccinated mice during eight week immunization period (n=4). Mice were immunized with either TBS (●), MVA (▽), FSME-IMMUN® (○) or MVA-prME (▲).


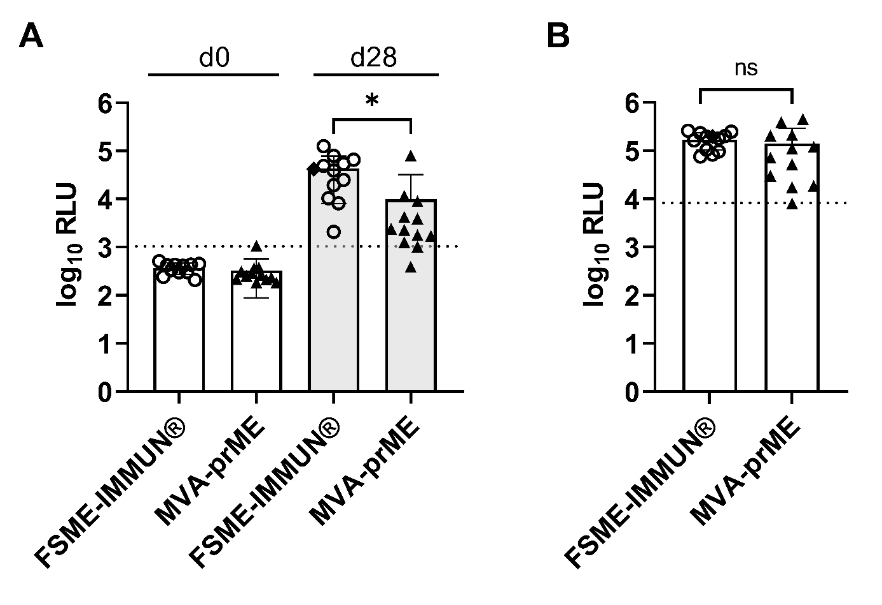


**Supplementary Figure 2**. TBEV EDIII-specific antibodies. (A, B) Sera of mice vaccinated with FSME-IMMUN® (○) or MVA-prME (▲) were tested in LIPS assay specific for domain III of the TBEV E protein. (A) Tested sera were collected before prime (d0, white bars) or before boost dose was administered (d28, grey bars) (n=1-3). (B) Sera were collected eight weeks after the prime vaccination (d56) (n=2-3). Luciferase activity is expressed as log_10_ relative light units (RLU) and presented are averaged values. Values above the average of naïve serum plus five-times its standard deviation are considered positive (dotted lines). The FSME-IMMUN®-vaccinated mouse that displayed signs of disease is highlighted with a diamond symbol. n.s.: not significant


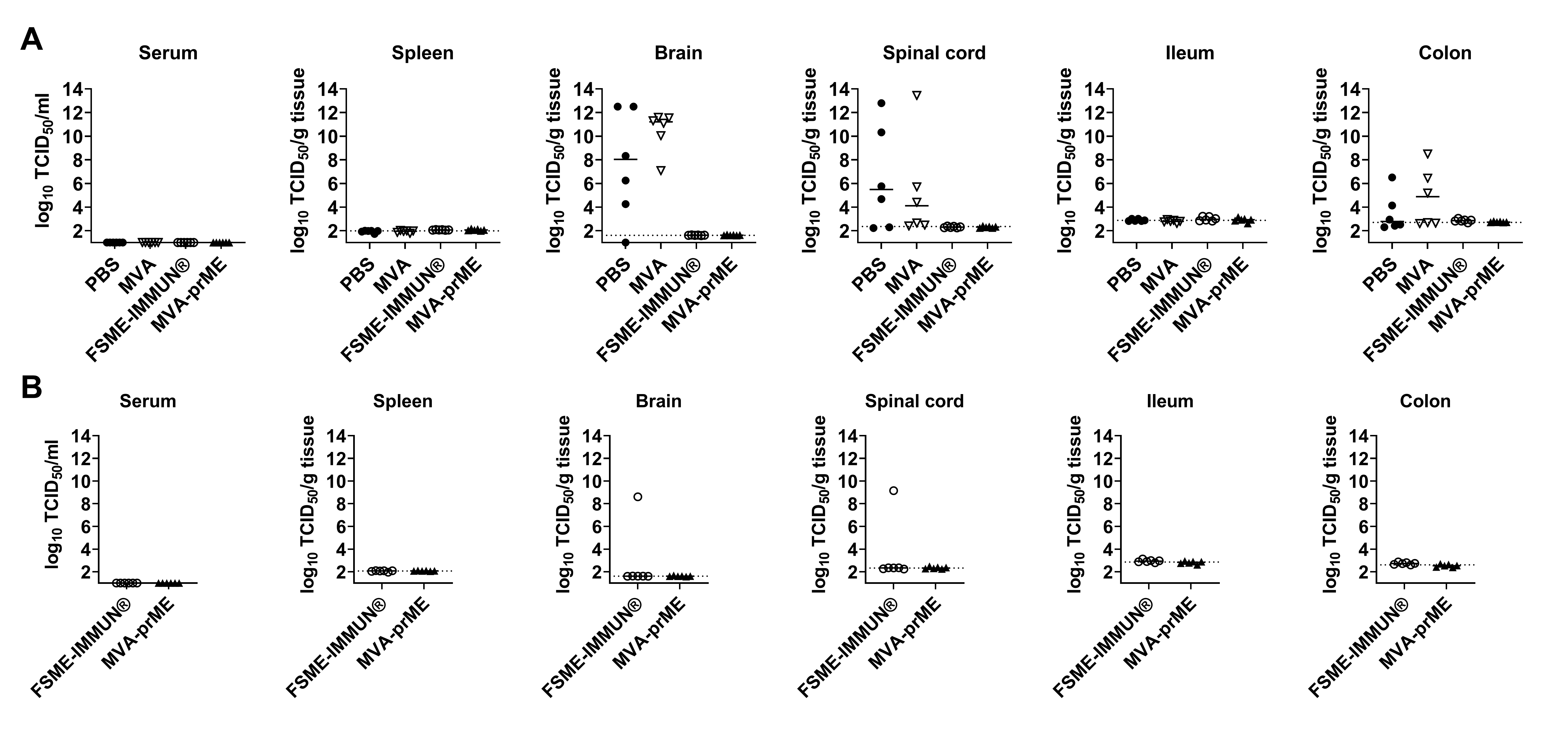


**Supplementary Figure 3.** Absence of infectious TBEV in periphery, CNS and GIT of mice vaccinated with MVA-prME or FSME-IMMUN®. Sera and cleared organ homogenates collected on day of sacrifice were tested for presence of infectious TBEV by TCID_50_ assay on A549 cells. Mice were either sacrificed at (A) 8 dpi or (B) stayed in the experiment until study endpoint (16 dpi). Titer of infectious TBEV is expressed as log_10_ TCID_50_ per ml or gram tissue. Dotted lines indicate detection limits for respective organs. Median is indicated in the graphs. Mice were immunized with PBS (●), MVA (▽), FSME-IMMUN® (○) or MVA-prME (▲).


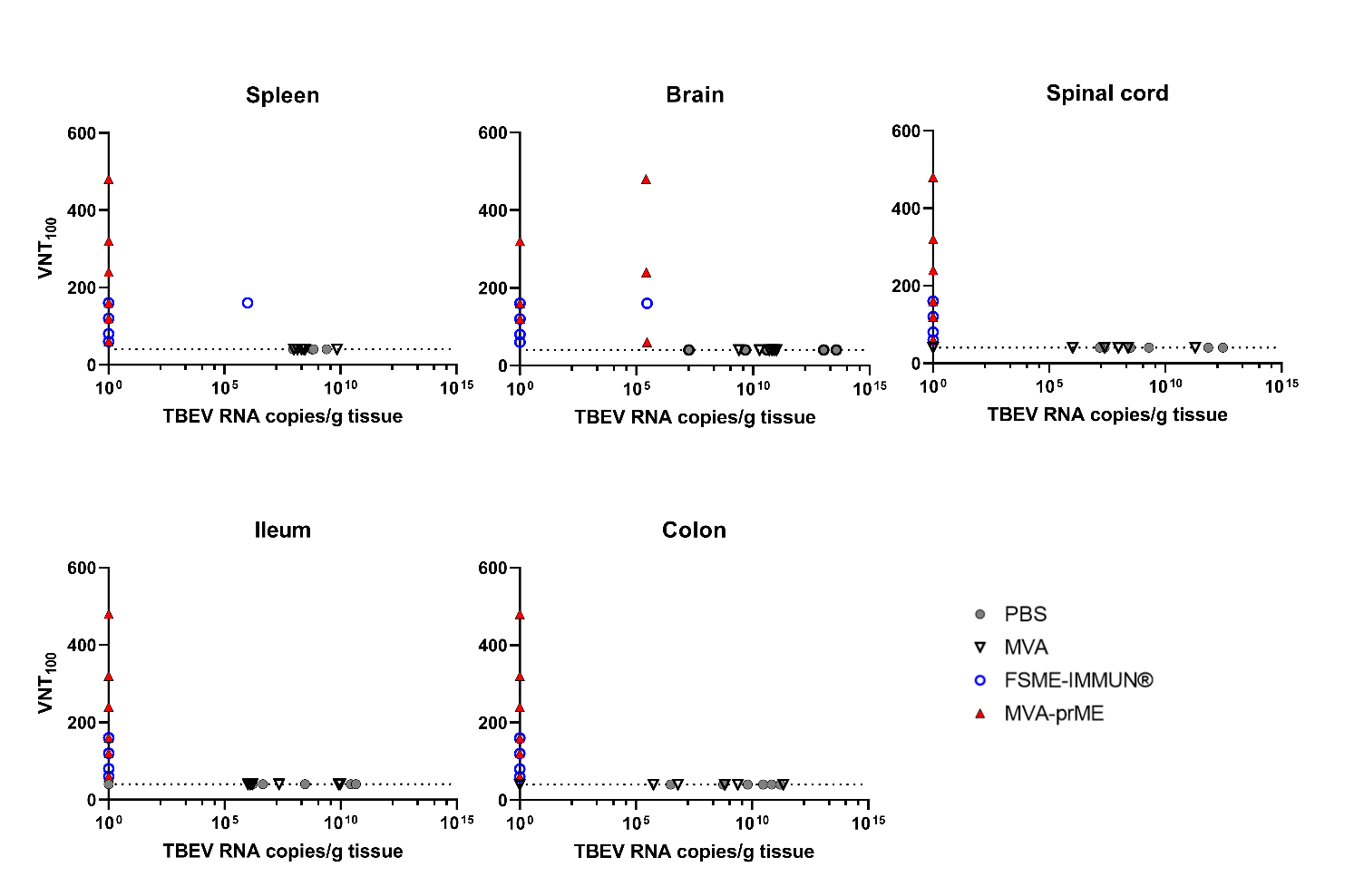
**Supplementary Figure 4.** VN antibody titers measured on day of TBEV challenge infection (VNT_100_) and TBEV RNA copy numbers measured at 8 dpi for spleen, brain, spinal cord, ileum and colon. Mice were immunized with PBS (gray circle), MVA (non-filled triangle), FSME-IMMUN® (non-filled blue circle) or MVA-prME (red triangle).
